# The Drunkard (Poetry)

**Published:** 1881-01

**Authors:** 


### THE DRUNKARD.

[Written for Hall's Journal of Health.]

With song and drunken shout,
Stag'ring the street about,
See ! see him come !
Striving in vain to find,
With his bewildered mind,
Way to his home.

See how he stares about
Hopelessly in his doubt ;
Unkempt his hair.
See how his ragged coat,
And all his garments float
In the keen air.

Watch how he reels along;
Hushed now his drunken song;
Sick and half blind,
Shunning once more a fall,
Reeling against a wall,
Support to find.

See where his footsteps tend,
See where his wand'rings end,
Aye, end forever.
There he stands on the brink,
Ready, alas ! to sink,
In the dark river.

Oh, by the love you bear
Your blue-eyed lad so fair,
Bid, bid him wait;
Reach forth a hand and save
The life the Father gave,
Ere 'tis too late.

Look at the droop of him,
Aye, and the stoop of him;
Vacant his eye.
See how he lifts his head,
Murmuring, "that I were dead,"
Toward the cold sky.

Oh, by the hopes you cherish,
Bid him ne'er to perish,
Nor yet despair;
Say there's a God above,
Bid him to trust His love,
Find strength in prayer.

Vain, vain; see him sink,
Over the dreadful brink,
With a sharp cry.
See, see him! struggling, rise
Lifting his staring eyes,
Still to the sky.

Once down a village street,
Pattered his little feet,
Joyous and gay;
Soft raiments covered him,
Tend'rest love hovered him,
Life was all May.

Clear were his happy eyes,
Blue as the summer skies;
Golden his hair,
Tossing in rings about,
The while his merry shout,
Filled all the air.

Pride of the village school,
Keeper of every rule,
Gen'rous and kind;
Joy of his mother's heart,
(Joy, how vain thou art!)
Manly, refined.

Quick, to his rescue go,
Lift him up gently; so.
Ah ! he is dead.
Close up the sightless eyes,
Filled with a vague surprise,
Cover his head.

And, ere you leave him there,
Say o'er a bit of prayer,
Tender and low,
E'en that his soul, set free
From the soiled clay, may be
Pure as the snow. —F. J. S.

BEESWAX and salt will make flat-irons as clean and as smooth as glass. Tie a lump of wax in a rag, and keep it for that purpose ; when the irons are hot rub them with the wax-rag, then scour with a clean paper or cloth sprinkled with salt.

TO MEND broken crockery, use lime and the white of an egg. Mix only enough to mend one article at a time, as it soon hardens, when it cannot be used. Powder a small quantity of the lime, and mix to a paste with the egg. Apply quickly to the edges, and place firmly together. It will soon become set and strong, seldom breaking in the same place.

